# Challenges in the management of familial hypercholesterolemia: a case report

**DOI:** 10.3389/fcvm.2024.1417432

**Published:** 2024-09-17

**Authors:** Joanna Rogozik, Marcin Grabowski, Renata Główczyńska

**Affiliations:** First Department of Cardiology, Medical University of Warsaw, Warsaw, Poland

**Keywords:** case report, familial hypercholesterolemia, inclisiran, LDLR gene, PCSK9

## Abstract

**Background:**

Familial hypercholesterolemia (FH) is a serious genetic condition that results in abnormally high levels of low-density lipoprotein cholesterol (LDL-C) in the bloodstream, significantly increasing the risk of early onset of cardiovascular disease. The heterozygous form of FH (HeFH) is widespread, affecting around 1 in 500 people worldwide.

**Case report:**

In this clinical report, we present the case of a patient who suffers from HeFH due to a mutation in the LDL receptor (LDLR) gene. A woman exhibited intolerance to statin therapy and did not attain adequate reduction in low-density lipoprotein cholesterol (LDL-C) levels on ezetimibe monotherapy. Genetic testing confirmed the presence of a pathogenic variant for FH with the deletion of exons 7–14. The administration of alirocumab (a dose of 150 mg sc) as the primary therapy did not exhibit the desired therapeutic outcome. Consequently, the patient was given inclisiran therapy (a dose of 284 mg sc), which significantly reduced LDL cholesterol levels after 3 months of treatment and during the 1-year follow-up.

**Conclusion:**

Inclisiran therapy has shown promising results for individuals with HeFH who experience statin intolerance. This therapy works by using a small interfering RNA (siRNA) to target the mRNA of proprotein convertase subtilisin/kexin type 9 (PCSK9), which leads to a significant reduction of LDL-C levels. This approach can be an alternative for patients without significant reductions in LDL-C levels with PCSK9 inhibitor therapy. For HeFH patients with limited treatment options due to statin intolerance and genetic mutations, inclisiran can represent a promising therapeutic option.

## Introduction

Familial hypercholesterolemia (FH) is a severe genetic disorder that leads to high levels of low-density lipoprotein cholesterol (LDL-C) in the blood and increases the risk of early onset of cardiovascular diseases (CVD) (1). It is an autosomal codominant disease and the most common form of monogenic hypercholesterolemia (2, 3). Cholesterol-lowering therapies have been found to reduce the risk of mortality and major cardiovascular events in individuals with FH. The heterozygous FH (HeFH) form is quite prevalent, with 1 in 500 individuals affected worldwide (4, 5). The estimated prevalence of FH in Poland is 1 in 250 individuals (6, 7).

According to the latest guidelines on lipid-lowering treatment of the European Society of Cardiology (8), patients with FH who have not achieved target LDL-C levels should be treated with proprotein convertase subtilisin/kexin type 9 (PCSK9) inhibitors. This group includes human monoclonal antibodies, i.e., alirocumab and evolocumab, by inhibiting the binding of PCSK9 to low-density lipoprotein receptor (LDLR), increases the number of available LDL receptors thereby reducing LDL-C levels from the circulation (9–14). Inclisiran is a novel small interfering RNA (siRNA) that is administered subcutaneously and works by inhibiting the synthesis of PCSK9 in the liver. This mechanism leads to a significant reduction in circulating LDL-C levels. Inclisiran is a first-in-class drug that has shown high effectiveness as a treatment for patients with hypercholesterolemia (15).

This study presents a case report of a woman with HeFH triggered by a mutation in the LDLR gene. The patient had a complete intolerance to statins, which led to the decision to initiate alirocumab therapy. Unfortunately, the therapy turned out to be ineffective. However, the latest treatment method with inclisiran was implemented which resulted in a significant reduction of LDL levels and the risk of adverse cardiovascular events.

## Case report

### Patient information and clinical findings

In December 2021, a 42-year-old woman was referred to our Outpatient Lipid Clinic (University Clinic Center of the Medical University of Warsaw) for the management of FH. The individual had a genetically confirmed mutation in the LDLR gene and had a family history with documented premature CVD. Carotid ultrasound revealed the presence of atherosclerosis plaques. On physical examination, the patient had corneal arcus, a typical finding in severe hypercholesterolemia. The patient's hypercholesterolemia was found to have no underlying secondary causes such as diabetes mellitus, thyroid gland disorders, renal or hepatic dysfunctions, or hypertension. Additionally, the patient was not taking any systemic corticosteroids or estrogens. In Table 1 we presented patient characteristics based on Dutch Lipid Clinic Network.

**Table 1:** Patient characteristics based on Dutch Lipid Clinic Network.

Dutch lipid clinic network criteria	Patient's personal history	Points
Family history		
First-degree relative with premature coronary or vascular disease, or a first-degree relative with LDL-C >the 95th percentile	Yes	1
	Father had MI at 40 years of age	
	Grandmother had a stroke at 60 years old	
First-degree relative with tendinous xanthomata and/or arcus corneal, or children aged <18 years with LDL-C >95th percentile	Yes	**2**
	Son with confirmed FH	
Clinical history		
Patient with premature CAD	No	2
Patient with premature vascular disease	No	1
Physical examination		
Tendinous xanthomata	No	6
Arcus corneal before age 45 years	Yes 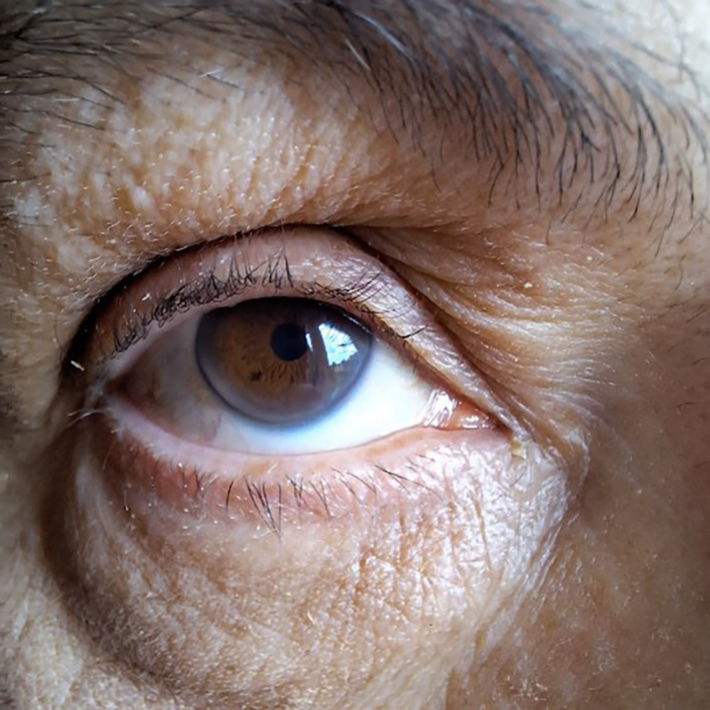	**4**
LDL-C levels (without treatment)		
LDL-C ≥8.5 mmol/L (≥325 mg/dL)		8
LDL-C 6.5–8.4 mmol/L (251–325 mg/dL)		5
LDL-C 5.0–6.4 mmol/L (191–250 mg/dL)	LDL-C 250 mg/dL	**3**
LDL-C 4.0–4.9 mmol/L (155–190 mg/dL)		1
DNA analysis		
Functional mutation in the LDLR, apoB, or PCSK9 genes	Yes	**8**
	LDLR 7–14 exon deletion	
A “definite” FH diagnosis requires >8 points	Definitive diagnosis	**17**

Points where the patient met the DCLN criteria and the summary score are highlighted in bold.

APOB, apolipoprotein B; CAD, coronary artery disease; FH, familial hypercholesterolemia; LDL-C, low-density lipoprotein cholesterol concentration; LDLR, low-density lipoprotein receptor; MI, myocardial infarction.

Previously, the patient was administered atorvastatin and rosuvastatin, leading to myalgia and elevated creatine kinase (CK) levels. The patient's intolerance to statins was confirmed, and during ezetimibe monotherapy, the patient did not achieve the desired therapeutic outcome. In the absence of any lipid-lowering treatment, the patient had the following lipid values: total cholesterol (TC) 315 mg/dL, non-HDL cholesterol (n-HDL) 250 mg/dL, high-density lipoprotein cholesterol (HDL-C) 65 mg/dL, triglycerides (TG) 58 mg/dL and LDL-C 238 mg/dL (calculated using Friedewald formula).

### Genetic analysis

The Dutch Clinic Lipid Network (DLCN score)—17 points indicated that the patient had a definite clinical diagnosis of HeFH. The genetic using direct sequencing and MLPA techniques test confirmed the presence of a pathogenic variant for FH, classified as c.2311 + 36G > C/p. (deletion of exons 7–14 of the LDLR gene in heterozygosis). This particular variant has a 50% chance of being passed down to first-degree relatives.

### Therapeutic intervention

**Figure 1:**
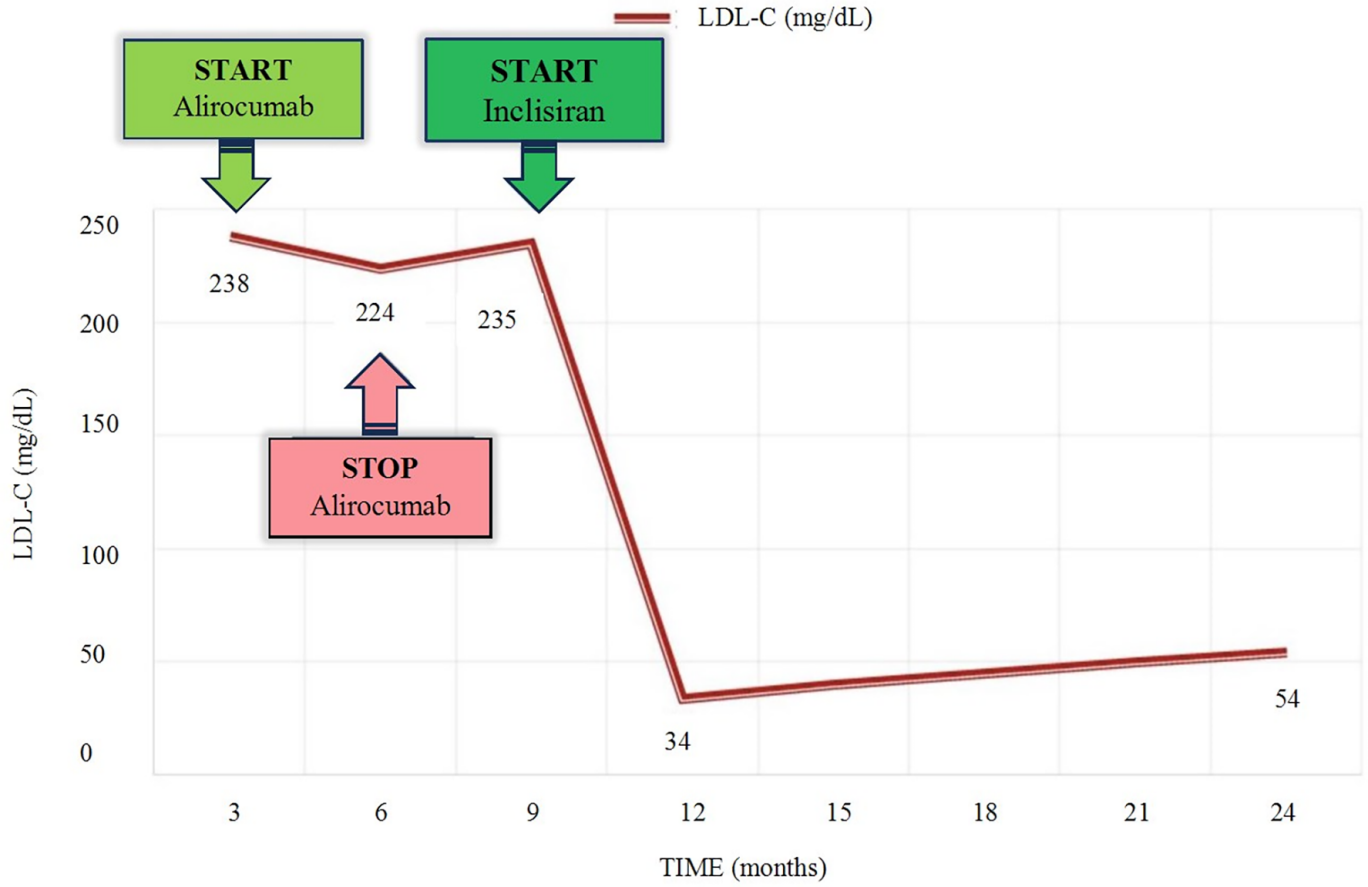
Timeline of LDL-C level variations according to lipid-lowering therapy. LDL-C, low-density lipoprotein cholesterol.

The patient had met the necessary criteria in the Polish FH treatment program to receive alirocumab, which was administered subcutaneously at a dose of 150 mg every 2 weeks. The woman underwent clinical evaluation and lab test follow-up after 3 months of treatment. Alirocumab was well-tolerated. No adverse effects were reported. The results of the laboratory tests showed that there was no significant decrease in LDL cholesterol. After unsuccessful treatment with a PCSK-9 inhibitor, the decision was made to begin administering inclisiran therapy. The patient was given 284 mg of inclisiran subcutaneously and follow-up tests were conducted after 3 months. The results showed a significant decrease in LDL cholesterol along with detailed lipid profile parameters: TC 114 mg/dL, non-HDL at 45 mg/dL, HDL-C at 69 mg/dL, TG at 53 mg/dL and LDL-C at 34 mg/dL. The next drug administration was scheduled in 6 months. Following a year of administering inslisiran, a noticeable positive therapeutic effect with LDL-C 54 mg/dL has been consistently observed and maintained. Individual LDL-C values over time are illustrated in Figure 1.

## Discussion

FH is primarily caused by loss-of-function mutations in the gene responsible for encoding LDLR, apolipoprotein B (APOB) genes, or gain-of-function mutations in the PCSK9 gene. These mutations are observed in over 90% of patients (16). Typically, HeFH is caused by a single pathogenic variant in one of three main genes: LDLR, APOB, and PCSK9 (17). Although genetic testing is the most reliable way to diagnose FH, it is not always available or affordable. However, clinical scores based on criteria such as the DCLN score, WHO, Simon Broome Register, MEDPED, Montreal-FH-Score (18), and JAS FH criteria (19) can be used to diagnose FH without genetic testing. These criteria help classify FH as certain, probable, or possible (18, 20, 21). Early diagnosis and treatment of FH is crucial to normalize life expectancy.

In this research, we present a case study of a patient diagnosed with HeFH resulting from a mutation in the LDLR gene. In monogenic FH, the LDLR gene is found to be dysfunctional in 60%–90% of cases, leading to impaired LDL clearance from the bloodstream (17, 22). The LDL receptor gene is located on the short arm of chromosome 19 (19p13.2) and consists of 18 coding regions (exons).

The American College of Medical Genetics and Genomics (ACMG) introduced an algorithm in 2015 that categorizes all variants of LDLR gene mutations into five groups: pathogenic, likely pathogenic, variant of unknown significance (VUS), likely benign, and benign (23, 24). Deletions encompassing exon 7–14 within the LDLR gene identified in the patient based on the algorithm mentioned above are classified as pathogenic.

The patient under consideration has a confirmed mutation in the LDLR gene, with a heterozygous variant. In addition, she has been diagnosed with complete statin intolerance (SI). Discontinuation of statin therapy is most frequently caused by statin-associated muscle symptoms (SAMS), which are also the most common adverse effects of statins (25, 26). Other potential statin-related adverse effects include neurocognitive disorders, hepatotoxicity, haemorrhagic stroke, and renal toxicity (27, 28). The issue of statin intolerance and the resulting discontinuation of statin therapy is a persistent clinical challenge that is prevalent on a global scale (29, 30). According to the subgroup analysis of a 2022 meta-analysis titled “Prevalence of statin intolerance”, the prevalence of statin intolerance (SI) was found to be 9.0% in primary prevention patients with FH (31).

Alirocumab, a monoclonal antibody that targets protease PCSK9, has proven to be highly effective in patients with atherosclerotic CVD and/or HeFH who require further reduction in LDL-C levels. Studies have shown that alirocumab can achieve reductions of between 55% and 60% in LDL-C levels in such patients (32). In a recent clinical trial, ODYSSEY OUTCOMES, it was found that the use of alirocumab, a PCSK9 inhibitor, significantly reduced the risk of major adverse cardiovascular events (MACE) when compared to a placebo (33). Non-responsiveness to human PCSK9 monoclonal antibodies is exceedingly low. However, in the rare cases where non-responsiveness does occur, clinicians are concerned about the potential presence of anti-drug antibodies. Phase 3 ODYSSEY studies examined apparent hyporesponsiveness to alirocumab, defined as <15% LDL-C reduction from and was reported in 1% of the patient study population appeared to be due to lack of adherence to therapy, a theoretical and rare possibility of biological non-responsiveness due to persistent antidrug antibodies, or other causes, as yet unidentified (34).

Alirocumab was found to be highly tolerable in our patient. According to the patient's self-report, all prescribed doses were administered as scheduled following prior training, and no reported adverse effects. In the study, inclisiran was administered by healthcare professionals on site, while patients self-administered four out of six doses of alirocumab at home. Nonadherence to the prescribed dosage regimen may have contributed to the observed lack of treatment response.

Inclisiran is a novel small interfering RNA (siRNA) that selectively targets the liver and suppresses the translation of PCSK9, a protein that regulates cholesterol metabolism. This leads to increased recycling of LDL receptors, which in turn increases the uptake of LDL-C and reduces its levels in the bloodstream. In clinical trials known as ORION, inclisiran has shown significant efficacy in reducing LDL-C levels when used as an adjunct to maximally tolerated statin therapy, specifically in patients with HeFH (15, 35, 36). On the 510th day of the study, it was observed that 99% of all patients who were enrolled and administered inclisiran achieved a significant 39.7% reduction in LDL-C levels (15). In the presented case the attainment of the therapeutic goal became possible solely due to the application of insclisiran. The present state of the patient is stable, and she gave her consent to proceed with the inclisiran therapy.

## Conclusions

In the case of a patient with HeFH, the process of selecting a suitable treatment option posed a considerable challenge due to their inability to tolerate statins and inadequate response to alirocumab. This rare condition is not commonly encountered in clinical practice, making it a complex case to manage effectively. However, with the use of inclisiran, a modern therapy, it was possible to effectively lower LDL-C levels and significantly decrease the patient's cardiovascular risk.

## Data Availability

The raw data supporting the conclusions of this article will be made available by the authors, without undue reservation.
